# Atrial fibrillation and cycling: six year follow-up of the Taupo bicycle study

**DOI:** 10.1186/s12889-014-1341-6

**Published:** 2015-01-21

**Authors:** Alistair Woodward, Sandar Tin Tin, Rob N Doughty, Shanthi Ameratunga

**Affiliations:** Section of Epidemiology and Biostatistics, School of Population Health, University of Auckland, Auckland, New Zealand; National Institute of Health Innovation and Department of Medicine, University of Auckland, Auckland, New Zealand

**Keywords:** Cycling, Cardiac arrhythmias, Physical activity, Atrial fibrillation

## Abstract

**Background:**

Atrial Fibrillation (AF) is the most common sustained cardiac arrhythmia, and the incidence of AF is increased markedly among elite athletes. It is not clear how lesser levels of physical activity in the general population influence AF. We asked whether participation in the Taupo Cycle Challenge was associated with increased hospital admissions due to AF, and within the cohort, whether admissions for AF were related to frequency and intensity of cycling.

**Methods:**

Participants in the 2006 Lake Taupo Cycle Challenge, New Zealand’s largest mass cycling event, were invited to complete an on-line questionnaire. Those who agreed (n = 2590, response rate = 43.1%) were followed up by record linkage via the National Minimum Health Database from December 1 2006 until June 30 2013, to identify admissions to hospital due to AF.

**Results:**

The age and gender standardized admission rate for AF was similar in the Taupo cohort (19.60 per 10,000 per year) and the national population over the same period (2006-2011) (19.45 per 10,000 per year). Within the study cohort (men only), for every additional hour spent cycling per week the risk changed by 0.90 (95% confidence interval 0.79 – 1.01). This result did not change appreciably after adjustment for age and height.

**Conclusions:**

Hospital admission due to AF was not increased above the national rate in this group of non-elite cyclists, and within the group the rate of AF did not increase with amount of cycling. The level of activity undertaken by this cohort of cyclists was, on average, not sufficient to increase the risk of hospitalization for AF.

## Background

Atrial fibrillation (AF) is the most common sustained cardiac arrhythmia, and is increasing in frequency in many countries [1]. In Australia, annual admissions to hospital due to AF now exceed those resulting from heart failure, and are converging on admissions for acute myocardial infarction [2]. The costs of treatment are high, and the risks of subsequent morbidity and mortality are considerable. In Sweden, mortality was more than five times the population average in the three years after first admission to hospital for AF. Even amongst relatively young patients (35-64 years) without significant co-morbidity, the mortality rate was twice that expected [3].

It is 15 years since the first study linking AF with high intensity physical activity was published, [4] and it is well-known that the condition is more common among elite athletes. One systematic review and meta-analysis estimated the risk of AF in athletes was more than 5 times that in the general population [5]. The mechanisms are not fully understood, but may include atrial enlargement, increased vagal tone and myocardial fibrosis [6]. What is less certain is the effect of physical activity on the frequency of arrhythmias in the general population. A systematic review published in 2013 found four cohort studies with information relevant to the question and sufficiently detailed for a pooled analysis, and on the basis of these data the authors concluded that regular physical activity in the general population was not associated with an increased risk of AF [7]. In this analysis the combined Odds Ratio for AF when comparing the most physically active with the least active group was 1.08 (95% CI 0.97-1.21).

Interpreting the findings from the general population studies is difficult for many reasons, including the diversity of measurements of physical activity, the wide range of activities included (from slow walking to intense physical labour), the wide range of duration of athletic training, indications that the effect of activity on AF differs between men and women, and differences in the way that confounding and intermediate variables are handled in analysis. Independent risk factors for AF such as age, sex and height should be adjusted for in statistical models. Other factors that are known to influence AF incidence, such as high blood pressure, diabetes and body weight, may lie on the causal pathway between physical activity and AF, and should be treated differently.

There may be a U shaped relation between physical activity and AF, since those who undertake moderate amounts of exercise have fewer events than those who are sedentary [8]. This benefit may be mediated by the tendency of physical activity to lower blood pressure, moderate weight gain and improve glucose control. However even in the general population there are signs of increased risk of AF in some of the groups that are most active [9].

While it does appear that there is a relationship between occurrence of AF and elite endurance athletic training, the relationship between more modest athletic training and AF is less certain. Given the many positive effects of physical activity on wellbeing and the overall reduction in mortality [10] and the understandable reluctance of clinicians and public health practitioners to discourage people from being more active, more nuanced analyses are required. This is especially pertinent at a time when the number of non-elite participants in endurance events such as road running, multi-sports and long distance cycling is growing. For instance, in the United States the number of finishers in running events increased 80% between 2000 and 2012, and the number and variety of these events has grown year on year. (www.runningusa.org) Marathons, ironman competitions and multi-day cycling races were once regarded as extreme sports, and were the domain of a small number of highly trained competitors. However, these events now attract large numbers of men and women, few of whom would regard themselves as elite athletes.

We report here on the experience of participants in the Taupo Cycle Challenge, the largest road cycling event in New Zealand, which is promoted widely and includes cyclists with a great range of experience. Our objective was to determine whether the rate of admission to hospital due to AF and other arrhythmias was higher amongst participants in the Taupo Cycle Challenge than in the New Zealand population at large. We aimed to learn also whether, within the Taupo cohort, hospital admission due to AF was related to the frequency and intensity of cycling.

## Methods

### Design, setting and participants

The sampling frame comprised cyclists, aged 16 years and over, who enrolled online in the Lake Taupo Cycle Challenge. This is held each November on a 160 km course around Lake Taupo in the North Island of New Zealand and attracts about 10,000 participants. Categories include multi-lap races, a solo single-lap event, and a relay (4 riders, 40 km each). The Classic one lap race is restricted to cyclists with a racing licence (typically 50-100 riders), but the event is otherwise open to all, regardless of age, experience or cycling ability.

We recruited the majority of participants at the time of the 2006 event, as described, in detail, elsewhere [11,12]. Briefly, we sent email invitations, containing a hyperlink to the study information page, to 5653 entrants who provided their email addresses at registration for the event. At the bottom of the page, entrants could click a button agreeing to take part in the study. (The ethics committee did not require parental approval for those aged 16 years and over.) Those who agreed to take part filled in a web questionnaire that included age, gender, education, ethnicity, place of residence, height and weight, general cycling activity and experience of cycle crashes in the past twelve months. The questionnaire was completed and submitted by 2438 cyclists (43.1% response rate). We recruited another 190 cyclists from the 2008 event by including a short description about the study in the event newsletter. We re-surveyed all participants in December 2009 using a web questionnaire containing similar questions to those in the baseline questionnaire. Altogether 1537 participants (59% of all those who entered the study) completed the 2009 questionnaire. Finishing times were obtained from the results pages of the Lake Taupo Cycle Challenge website http://cyclechallenge.com/past-results/.

We obtained ethical approval for this study from the University of Auckland Human Participants’ Ethics Committee.

### Hospital discharge data and mortality records

The National Minimum Health Database, maintained by the New Zealand Ministry of Health holds information on inpatients and day patients discharged after a minimum stay of three hours from all public hospitals and over 90% of private hospitals in New Zealand [13]. The Mortality Collection Database includes information on all deaths registered in the country [14].

Participant data were matched to a National Health Index (NHI) number, a unique identifier assigned to every person who uses health and disability support services in New Zealand. An electronic match was made where possible, followed by two stages of manual matching for participants who could not be linked electronically. Of 2590 participants who were resident in New Zealand at recruitment, 99.0% were successfully matched.

All hospital discharges in the cohort, from December 1 2006 until April 30 2013 were extracted. The hospital discharge data contain diagnoses and diagnostic and therapeutic procedures undertaken in each hospital visit, coded using the ICD-10-AM classification system. Admissions for arrhythmias were identified from the codes I42 and I44-49. I48 is the key code for atrial arrhythmias, and it includes atrial fibrillation and also atrial flutter (a less common, related disorder that frequently precedes or accompanies fibrillation).

Readmissions were identified using an algorithm applied previously to New Zealand hospital data [15] and were excluded from the analysis.

### Analyses

The study sample was restricted to 2590 participants who were resident in New Zealand at recruitment. In follow-up, the participants were censored on 30 April 2013 or date of death. As more than a single event may be experienced, incidence rates of repeated events were calculated using the person-years approach. Confidence intervals were based on the Poisson distribution. For comparison with rates of hospital discharge in the national population, the Taupo cohort was restricted to those aged 15-69 years, and standardised for age and gender using the 2006 New Zealand census population as the standard. Aggregate hospital discharge data for the national population were obtained from the Ministry of Health website (http://www.health.govt.nz/nz-health-statistics/health-statistics-and-data-sets/hospital-event-data-and-stats) for the most recent period available (up to 30 June 2011). In this comparison we excluded the last 2 years of follow-up in the Taupo cohort to fit the time frame for national hospitalization data.

Cox proportional hazards regression modelling for repeated events was then performed using a counting process approach to assess hazards of AF associated with average time spent cycling. The robust sandwich estimates for the covariance matrix were used to account for the correlation among the events within subject. Hazard ratios (HRs) were adjusted for age and height (or, in an alternative analysis, for Body Mass Index). The within-cohort analysis was restricted to males due to the small number of events that occurred amongst females. A sensitivity analysis was undertaken using finishing time, another indicator of amount of cycling, as an exposure.

All analyses were performed using SAS (release 9.2, SAS Institute Inc., Cary, North Carolina).

## Results

The study cohort is predominantly male, non-Maori and relatively well-educated (Table 1). Between 2006 and 2013, there were eight deaths in the study group; the causes were cancer (5) and bicycle-car collision (1). Two cases are waiting for a coroner’s finding. In Table 2 we show the numbers of admissions to hospital for AF. Note that these constitute first admissions for the same event only. Of the 34 participants hospitalized due to AF during the follow-up period, 13 were admitted for more than one event (median 2, range 2-9).

**Table 1:** **Characteristics of the Taupo cycle challenge cohort**

	**N (%)**
**Age**	
16-34	504 (19.46)
35-49	1340 (51.74)
50+	746 (28.80)
**Gender**	
Male	1874 (72.4)
Female	725 (27.6)
**Highest education level**	
Secondary school or less	535 (20.7)
Polytech	654 (25.3)
University	1395 (54.0)
**Ethnicity**	
Maori	104 (4.0)
Non-Maori	2486 (96.0)
**Height (cm)**	
Less than 165	322 (12.5)
165-179	1295 (50.1)
180 or more	967 (37.4)
**Body Mass Index**	
Less than 25	1361 (52.9)
25-30	1000 (38.8)
30 or more	214 (8.3)
**Cycling experience (years)**	
0-2	1081 (41.9)
3-7	755 (29.3)
8 or more	743 (28.8)
**Hours spent cycling per week**	
0-2	459 (17.7)
3-5	955 (36.9)
6 or more	1176 (45.4)
**Km cycled per week**	
0-49	389 (15.1)
50-99	620 (24.0)
100 or more	1572 (60.9)
**Frequency of cycling per week**	
0-1	403 (15.6)
2-4	1694 (65.4)
5 or more	493 (19.0)

**Table 2:** **Hospitalizations for Atrial Fibrillation and Atrial Flutter, by age, gender and other known risk factors, in the Taupo Cycle Challenge cohort, 2006-2013**

	**No of events**	**Person-years**	**Rate per 10000 person-years**
	**66**	**16249.68**	**40.62 (31.41, 51.67)**
**Age**			
16-34	1	3154.68	3.17 (0.08, 17.66)
35-49	26	8419.21	30.88 (20.17, 45.25)
50+	39	4675.78	83.41 (59.31, 114.02)
**Gender**			
Male	63	11789.14	41.06 (68.37)
Female	3	4460.54	6.73 (1.39, 19.66)
**Height**			
Less than 165	2	1998.55	10.01 (1.21, 36.15)
165-179	19	8136.39	23.35 (14.06, 36.47)
180 or more	45	6080.18	74.01 (53.98, 99.03)
**Body Mass Index**			
Less than 25	24	8559.62	28.04 (17.96, 41.72)
25-30	35	6275.75	55.77 (38.85, 77.56)
30 or more	5	1323.89	37.77 (12.26, 88.14)

Re-admissions excluded.

We compared the frequency of hospital admissions for all cardiac arrhythmias in the Taupo cohort with national rates for the period 2006-2011 (Table 3). The age and gender standardized admission rate for AF was almost exactly the same in the Taupo cohort (19.60 per 10,000 per year) as in the national population (19.45 per 10,000). Rates for other arrhythmias were also similar to national figures, although comparisons are constrained by small numbers.

**Table 3:** **A comparison of frequency of hospital admissions for cardiac arrhythmias in the Taupo cohort with national rates, 2006-2011, 15-69 year olds only, 95% confidence intervals are included for the Taupo results**

	**Taupo data**					**National rate**
	**No**	**Person-years**	**Crude rate per 10000 person-years**	**Age standardised rate per 10000 person-years**	**Age & sex standardised rate per 10000 person-years**	
Any	57	11463.73	49.72 (37.66, 64.42)	56.84 (42.85, 70.82)	36.94 (25.56, 48.31)	31.49
Atrial fibrillation & flutter	38	11463.73	33.15 (23.46, 45.50)	33.75 (23.42, 44.08)	19.60 (11.72, 27.47)	19.45
Paroxysmal tachycardia	12	11463.73	10.47 (5.41, 18.29)	10.10 (3.85, 16.35)	9.19 (3.22, 15.17)	5.67
Other cardiac arrhythmia	3	11463.73	2.61 (0.54, 7.62)	2.84 (0, 5.78)	1.62 (0, 3.82)	2.64
AV and LBB block	3	11463.73	2.61 (0.54, 7.62)	2.56 (0, 5.45)	2.54 (0, 5.50)	1.06
Other conduction disorders	1	11463.73	0.87 (0.02, 4.84)	7.49 (1.79, 13.19)	4.91 (0.26, 9.56)	0.55
Cardiac arrest	2	11463.73	1.74 (0.21, 6.28)	1.43 (0, 3.65)	0.85 (0, 2.56)	0.25
Cardiomyopathy	2	11463.73	1.74 (0.21, 6.28)	1.15 (0, 3.31)	0.77 (0, 2.50)	1.87

The analysis of admissions for AF in relation to levels of cycling drew on information provided at entry to the study on frequency of cycling, time spent cycling per week, and distance ridden (Table 4).

**Table 4:** **Hospital admissions for Atrial Fibrillation and Atrial Flutter (AF) during 2006-2013 in relation to levels of cycling reported in 2006, Taupo Cycle Challenge cohort, males only**

	**Number admitted for AF**	**Person-years**	**Rate per 10000 person-years**
**Total**	**66**	**16249.68**	**40.62 (31.41, 51.67)**
***Hours spent cycling per week***			
0-2	8	2891.71	27.67 (11.94, 54.51)
3-5	34	6001.34	56.65 (39.23, 79.17)
6 or more	24	7356.63	32.62 (20.90, 48.54)
***Km cycled per week***			
0-49	5	2445.56	20.45 (6.64, 47.71)
50-99	27	3894.70	69.32 (45.69, 100.86)
100 or more	32	9853.56	32.48 (22.21, 45.85)
***Frequency of cycling per week***			
0-1	6	2544.24	23.58 (8.65, 51.33)
2-4	57	10640.04	53.57 (40.57, 69.41)
5 or more	3	3065.41	9.79 (2.02, 28.60)

There was no sign that admission rates were higher amongst the more active participants than those who cycled less. For every additional hour spent cycling per week, the risk reduced by 0.90 (95% confidence interval 0.79 – 1.01). After adjustment for age and height the hazard ratio remained 0.90 (0.79-1.03). The model was run separately to control for BMI, and again this did not change the association between time spent cycling and hospitalisation for AF. There was no evidence of modification by age (50 and over compared with under 50 years).

For 1751 participants who completed the solo, one lap event, we examined the relation of hospitalisation for AF and finishing time (minimum 3 hours 57 minutes; maximum 7 hours 45 minutes; median 5 hours 58 minutes). By quartile, fastest to slowest, the age- and gender-adjusted Hazard Ratios were 0.65 (0.09-4.48); 0.35 (0.06-2.26); 0.70 (0.14-3.44); and 1.0 (reference category).

## Discussion

Amongst participants in the Taupo Cycle Challenge study, the rate of first hospital admission for AF was almost identical to that for the adult New Zealand population at large, amongst whom about 50% do not meet the physical activity guideline of “at least 30 minutes of moderate-intensity physical activity on most if not all days of the week” [16]. Admissions for AF were strongly related to age, gender and height, as has been reported previously [7]. If the principal finding was due, at least in part, to health selection (whereby those with existing AF were discouraged from entering the event in 2006), one would expect the rate in the cohort to diverge from the national rate with increasing duration of follow-up. However this was not evident when we compared rates on a year by year basis, from 2006-8 to 2010-11.

Within the cohort, we compared levels of cycling, as reported at entry to the study in 2006, with subsequent admissions to hospital due to AF. Among males, there was no relation seen between the frequency or intensity of cycling and later risk of AF, a finding that did not shift after controlling for age, height and BMI. Indeed, while the estimate was imprecise (the 95% confidence interval included 1.0), our result suggested a protective effect for AF, rather than an increase in risk.

Strengths of this study, compared with many of those reported previously, include the longitudinal design, and the use of record linkage to ensure the complete and unbiased ascertainment of outcomes as defined by hospitalised events. Unlike previous studies that have tended to concentrate on elite sportsmen and sportswomen, this study included a wide range of enthusiasts, novices, and experienced social riders.

We do not claim that the study sample is representative of all who participated in the Taupo event, nor of New Zealand cyclists in general. What is material to this paper is whether the relation between the exposure (amount of cycling) and risk of AF is different in the study group than amongst the wider group of cyclists. We see no reason why this should be the case.

The study reports hospital admissions for all occurrences of AF, which is a heterogeneous group of conditions, and it has been proposed that some sub-types are particularly sensitive to levels of physical activity [17]. Moreover it is almost certain that the data presented here do not capture all occurrences of AF. For example, short self-limiting episodes of paroxysmal AF will often not result in hospitalisation. Thus, we cannot determine from this study the absolute incidence of AF among this population of cyclists. However, we see no reason why the proportion of arrhythmias leading to hospital admission should systematically differ between Taupo Cycle Challenge participants and the population at large. If socioeconomic status is related positively to ascertainment and treatment, then this would tend to inflate the study rate compared with the population at large. If better educated individuals were more likely to be treated out of hospital in private practice this would have the opposite effect. In any event, the rate of hospital admission attributed to AF amongst Taupo participants was similar to the national figure, and we can see no reason why ascertainment within the study cohort should be biased by level of physical activity.

A weakness of the study is the reliance on questionnaire data regarding amount of cycling, which are likely to be an imprecise measure of physical activity. As another indicator of amount of cycling, we used finishing time, as was reported by Andersen et al [18] in a study of cross-country skiers. Unlike Andersen et al, we found no sign of higher rates of hospitalization for AF amongst those with faster finishing times. Also, our information on physical activity was collected at just one point in time (entry to the study in 2006). Many of those taking part in the Taupo event are involved in other cycling events, and train regularly. On the other hand, some participants may not have sustained the levels of cycling that were undertaken prior to Taupo, and mis-measurement of this kind would tend to disguise an increase in AF rates with increasing activity in our analysis. However, amongst those who completed the second questionnaire in 2009 levels of cycling were relatively stable overall: 23% reported cycling more than in 2006, 44% reported the same, and 28% reported cycling less. Five per cent had stopped cycling.

We did not determine whether participants were suffering from a sustained arrhythmia of any kind at the time they entered the study. If some were already experiencing periods of AF, this may have discouraged them from riding as much as they would otherwise, and might explain a relatively high rate of hospitalization with AF in the low exercising group, early in follow-up. To test this possibility, we excluded hospital events in the first year of the study. In the new analysis the HR for every additional hour of cycling per week changed very little from 0.90 (0.79-1.01) to 0.88 (0.78, 1.00).

How to reconcile the mixed results reported in the literature? We propose there may be competing forces acting on the incidence of AF. Intense activity at a young age may provide the substrate for arrhythmias that appear later in life (possibly via atrial remodelling, increased autonomic tone and micro-fibrotic changes in the atrial wall), while vigorous activity in older years may tend to reduce occurrence of arrhythmias via other pathways (such as lowering blood pressure). In a large cohort of Swedish men Drca et al [19] found a positive association with AF of leisure time walking and cycling at age 30, but not at ages 15 or 50. The same study found a protective effect of walking and cycling more than an hour a day at older ages.

## Conclusions

We followed 2500 participants in a mass cycling event who reported cycling on average 128 km per week, for an average of 6.4 years, and compared admissions to hospital with AF with national rates. There was no sign of an increase in hospitalisation among those who participated in the Taupo Cycle Challenge. Within the study cohort, we did not see a relation between the volume of cycling training and the frequency of admission to hospital with AF. We intend to continue the follow-up of the cohort and will review the incidence of arrhythmias. On the basis of the data to hand we conclude the level of physical activity undertaken by this cohort of cyclists did not, on average, influence hospital admissions for AF.

## Abbreviations

AF: Atrial fibrillation
HR: Hazard ratio
NHI: National health index
